# Small-Molecule Fluorescent Probes for Detecting Several Abnormally Expressed Substances in Tumors

**DOI:** 10.3390/mi13081328

**Published:** 2022-08-16

**Authors:** Leilei Yao, Caixia Yin, Fangjun Huo

**Affiliations:** 1 Research Institute of Applied Chemistry, Shanxi University, Taiyuan 030006, China; 2 Key Laboratory of Chemical Biology and Molecular Engineering of Ministry of Education, Institute of Molecular Science, Shanxi University, Taiyuan 030006, China

**Keywords:** fluorescence imaging, tumor identification, small molecule

## Abstract

Malignant tumors have always been the biggest problem facing human survival, and a huge number of people die from cancer every year. Therefore, the identification and detection of malignant tumors have far-reaching significance for human survival and development. Some substances are abnormally expressed in tumors, such as cyclooxygenase-2 (COX-2), nitroreductase (NTR), pH, biothiols (GSH, Cys, Hcy), hydrogen sulfide (H_2_S), hydrogen sulfide (H_2_O_2_), hypochlorous acid (HOCl) and NADH. Consequently, it is of great value to diagnose and treat malignant tumors due to the identification and detection of these substances. Compared with traditional tumor detection methods, fluorescence imaging technology has the advantages of an inexpensive cost, fast detection and high sensitivity. Herein, we mainly introduce the research progress of fluorescent probes for identifying and detecting abnormally expressed substances in several tumors.

## 1. Introduction

Cancer is a form of malignancy. For a long time, human beings have endured a huge threat from malignant tumors. Therefore, the precise identification and detection of tumor malignancy and migration at the cellular level, so as to accurately distinguish between the tumors and the surrounding healthy tissues, is of vital significance for human survival and development [1,2,3,4]. Over many years, more and more research has contributed to developing methods for detecting tumors to obtain information on the diseased area, size as well as the severity of the tumor, causing profound changes in the discovery and treatment of malignant tumors. Currently, some tumor detection technologies include nuclear magnetic resonance imaging, computed tomography, histopathological diagnosis, microfluidic technology and others [5,6,7,8]. However, the above several methods have limitations such as large radiation, high cost and complex operation, so it is urgent to develop a new testing platform to promote the development of the field of tumor testing.

Compared with these methods, fluorescent imaging is an emerging technology, with the advantages of high sensitivity, low cost, small radiation, fast detection, and good specificity [9,10,11,12]. Fluorescent probes are mainly composed of two parts, a signal unit and a recognition unit, which can make the detected cells/tissues emit fluorescence when irradiated by a light source with a specific wavelength, thereby allowing the visualization of the lesion sites [13]. High-performance near-infrared (NIR) fluorophores have good clinical application potential because of the advantages of low penetration, low fluorescence background as well as low damage to biological samples [14,15]. By chemically modifying these fluorophores, it is possible to develop NIR fluorescent probes with improved performance, leading to improved photophysical and chemical properties, including higher sensitivity, better photostability, longer wavelengths, and good specificity. Therefore, fluorescent probes are extensively applied in the imaging of various diseases.

**Figure 1:**
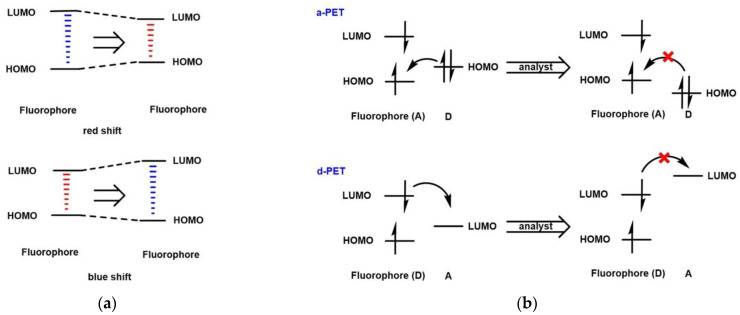

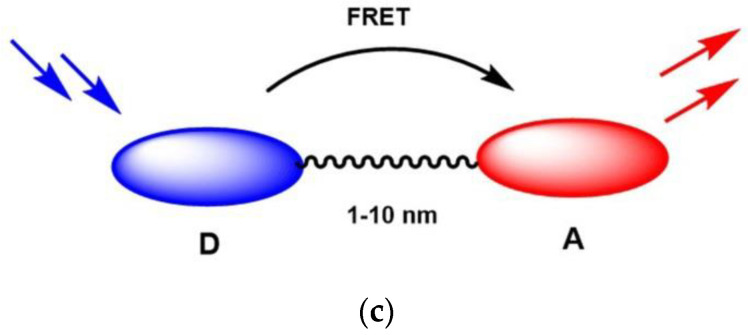
The mechanism of (**a**) ICT; (**b**) PET; (**c**) FRET.

The fluorescence regulation mechanisms of fluorescent probes mainly include intramolecular charge transfer (ICT), photo-induced electron transfer (PET), fluorescence resonance energy transfer (FRET), aggregation-induced emission (AIE), and excited-state intramolecular proton transfer (ESIPT), etc. ICT, PET, and FRET are commonly used in the design of fluorescent probes. ICT refers to the intramolecular charge transfer process that occurs when the probe is irradiated with excitation light. The probe designed based on the ICT mechanism contains an acceptor (A), a donor (D), and a conjugated structure connecting A and D. As shown in Figure 1a, when the electron-donating ability of D or the electron-withdrawing ability of A increases, ΔE_LUMO/HOMO_ decreases, and the fluorescence spectrum of the probe undergoes a red shift; otherwise, the fluorescence spectrum of the probe undergoes a blue shift. ICT can be used for the design of ratiometric fluorescent probes. PET refers to the process by which electrons are transferred from D to A when irradiated by excitation light. The PET-based probes are similar to ICT, except that A and D are linked in a non-conjugated structure. There are two types of PET: a-PET (fluorophore is A) and d-PET (fluorophore is D). As shown in Figure 1b, during the a-PET process, A is excited, electrons transition from HOMO to LUMO, and the electrons on the HOMO of D preferentially occupy the HOMO of A. Thus, electrons in the excited state of the fluorophore are prevented from returning to the ground state, resulting in the quenching of the fluorescence. After interacting with the analyte, the HOMO of D decreases, the above process is inhibited, and the fluorescence is activated. In the d-PET process, the LUMO of A is between the LUMO and HOMO of D, and the electrons of the fluorophore preferentially transition to the LUMO energy level of A after being activated, so that they cannot return to the ground state and the fluorescence is quenched. After the interaction with the analyte, the above process is interrupted and the fluorescence is activated. Most fluorescent probes of the PET mechanism are reversible. As shown in Figure 1c, the fluorescent probe based on the FRET mechanism has two fluorophores, one as an energy donor and one as an energy acceptor. It is worth noting that the emission spectrum of D and the absorption spectrum of A overlap. The excited state D transfers energy to A through energy resonance, so that A emits fluorescence with a longer wavelength. FRET is the most commonly used design mechanism for ratiometric fluorescent probes.

In recent years, several fluorescent probes for tumor imaging were successively developed [16,17]. However, the number of deaths due to malignant tumors has not decreased worldwide [18,19,20,21,22], and further research and development of novel probes for tumor identification and detection is urgent.

In the procedure of tumor growth, proliferation and migration, there will be metabolic changes different from normal cells. Many studies have shown that the expression of many substances in tumor cells/tissues is abnormal compared with that in normal cells/tissues. We can detect the position of tumor cells/tissues and the situation of malignancy based on the abnormal expression of these substances [23], for example, cyclooxygenase-2 (COX-2), nitroreductase (NTR), pH, GSH, other biothiols, H_2_S, H_2_O_2_, HOCl and nicotinamide adenine dinucleotide (NADH). In recent years, fluorescent probe imaging technology has made significant progress in this regard. In this review, we introduce the progress of small molecule fluorescent probes for detecting several abnormally expressed substances in tumors in recent years. We think that such a review would help more researchers devote themselves to this meaningful research field, so as to develop more substances for accurate detection, which is expected to enhance its value in clinical applications. We hope this review can provide a recent research status of small-molecule fluorescent probes detecting abnormally expressed substances in tumors for researchers in this field, and we look forward to continuous breakthroughs in this field.

## 2. Fluorescent Probe for Detecting Cyclooxygenase-2 (COX-2) Enzymes

Recent studies have found that cyclooxygenase-2 (COX-2) is closely related to the processes of tumor growth and metastasis [24,25,26]. Many data have shown that more than 60% of tumors can cause hypoxia [27,28,29,30,31], that COX-2 is highly expressed in tumors but less in normal cells [32,33], and the amount of COX-2 increases with tumor deterioration [34,35,36,37,38]. Therefore, the detection of COX-2 is of great value for identifying the tumor environment. At present, many probes have been developed for the detection of COX-2.

**Figure 2:**
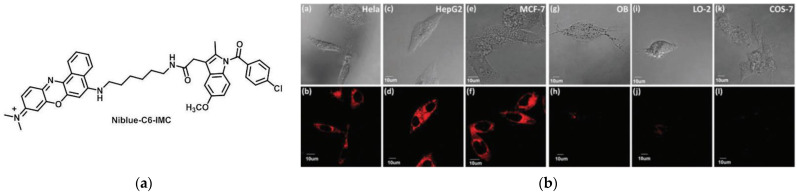
(**a**) Structure of probe Niblue-C6-IMC; (**b**) Confocal imaging of cancer cell lines (MCF-7 cells; HepG2 cells; HeLa cells) and normal cell lines (COS-7 cells; LO-2 cells; OB cells) staining with Niblue-C6-IMC. Reproduced with permission from [39]. Copyright 2015 The Royal Society of Chemistry.

In 2015, Peng et al. [39] introduced the first NIR fluorescent probe, Niblue-C6-IMC (Figure 2a), to localize COX-2 in the Golgi apparatus, which indomethacin (IMC) was linked to the Nile blue dye using a hexane diamine. The calculated results of Gaussian 09 showed the existence of PET between Nile blue dye and IMC. Hence, the fluorescence disappeared. When bound to COX-2, PET was suppressed, consequently, fluorescence was restored. By Native-PAGE analysis, the data showed that Niblue-C6-IMC can be specifically conjugated to COX-2. Then, cancer cell lines (HeLa cells; HepG2 cells; MCF-7 cells) and normal cell lines (COS-7 cells; LO-2 cells; OB cells) were subjected to a confocal fluorescence microscope (Figure 2b). The results indicated that Niblue-C6-IMC was capable of distinguishing normal cells from cancer cells. Colocalization experiments showed that the probe could efficiently mark the Golgi in cancer cells. Further, it could perform fluorescence imaging in tumor tissues and mice tumor sites. The probe is a powerful tool in the study of cancer procession.

**Figure 3:**
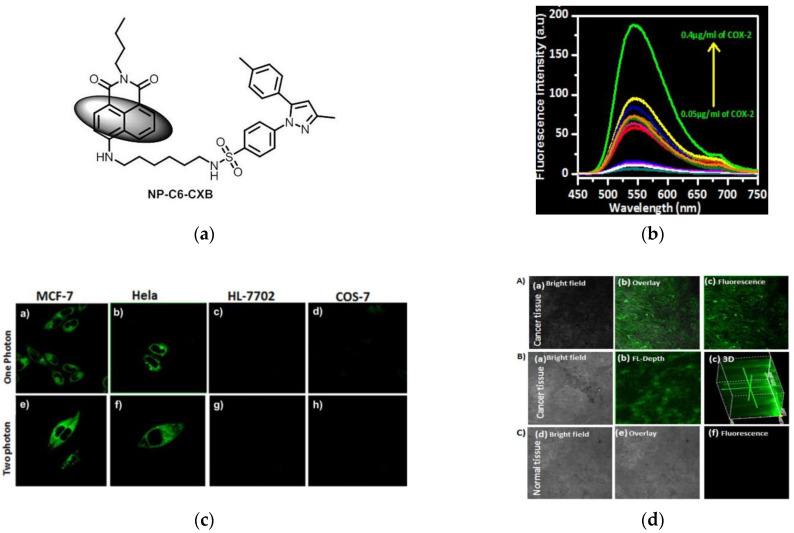
(**a**) Structure of probe NP-C6-CXB; (**b**) Fluorescence spectra of NP-C6-CXB in the presence of COX-2; (**c**) Fluorescent images of live cells under excitation of one-photon and two-photon; (**d**) Fluorescence imaging of cancer and normal tissues. Reproduced with permission from [40]. Copyright 2018 American Chemical Society.

Later, in 2018, Peng and coworkers [40] designed a one- and two-photon fluorescence probe NP-C6-CXB (Figure 3a). Naphthalimide was chosen as the fluorophore and celecoxib was chosen as the selection group for COX-2. NP-C6-CXB was in a PET forbidden state in solution, when interacted with COX-2, the PET recovered with a strong fluorescent response (Figure 3b). In living cells imaging, compared the fluorescence responses of cancer cell lines and normal cell lines, Figure 3c found that cancer cell lines (MCF-7 and Hela cells) fluoresced strongly, while normal cell lines (HL-7702and COS-7 cells) fluoresced weakly. Further, in the tissue slices imaging (Figure 3d), strong fluorescence was found in tumor tissue of Balb/c nude mouse, but not observed in normal liver tissue. Subsequent mouse experiments also proved this. This probe has potential value in identifying tumors.

**Figure 4:**
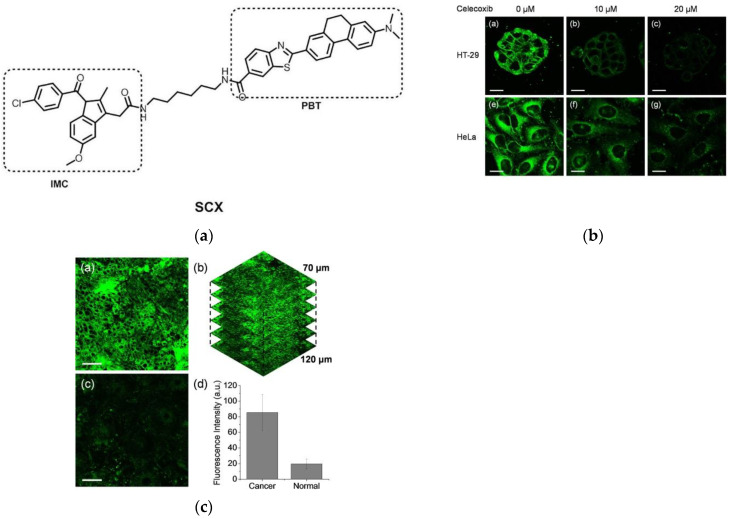
(**a**) Structure of probe SCX; (**b**) Two-photon fluorescence images of cells; (**c**) Two-photon fluorescence images of human colon (**c-a**) cancer tissues and (**c-c**) normal tissues. Reproduced with permission from [41]. Copyright 2020 Elsevier B.V.

In 2021, Kim’s research group [41] developed a novel two-photon fluorescent probe SCX (Figure 4a), which is based on the PBT fluorophore, and IMC was selected as the targeting group. In the imaging of HeLa cells, the probe emits distinct fluorescence under excitation at 810 nm. After pretreatment with the COX-2 inhibitor celecoxib in HT-29 and HeLa cells, the fluorescence intensity of both cancer cells was obviously reduced. Afterward, control experiments with cancer cell lines and normal cell lines (Figure 4b) were performed. HT-29 (colorectal adenocarcinoma), Huh-7 (hepatocellular carcinoma), HeLa (epithelioid cervical carcinoma) cells were selected as cancer cell lines, CCD-18Co (normal colon cells), Chang (normal hepatocytes), Raw264.7 (macrophages) was selected as the normal cell line. The results demonstrated that the fluorescence intensity of cancer cell lines and normal cell lines was significantly different. Further, colonic normal and tumor tissues were studied (Figure 4c). The results show that in cancerous tissue, SCX will emit strong fluorescence, and the observation depth can reach at least 100μm, which is 4.4 times the intensity of normal tissue. The above studies show that the probe has far-reaching significance for distinguishing normal tissue from cancerous tissue in living human samples.

**Figure 5:**
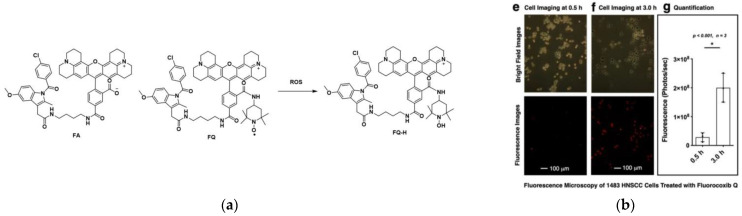
(**a**) Structure of FA, FQ, FQ-H; (**b**) Fluorescence images of 1483 HNSCC cells treated with FQ. Reproduced with permission from [42]. Copyright 2022 American Chemical Society.

In 2022, Marnett and co-workers [42] designed a redox-activatable probe, FQ, which was used to detect COX-2. FQ was obtained by linking FA to an amino-TEMPO molecule, which was converted to FQ-H when FQ was reduced (Figure 5a). FQ showed strong fluorescence after being converted into FQ-H, and then the detection of COX-2 can be realized. A total of 1483 HNSCC cells with high COX-2 expression were chosen for cell experiments (Figure 5b). The results showed that strong fluorescence appeared after FQ was incubated with cells for 3 h. Furthermore, in vivo experiments showed that FQ had sufficient time to reach the target in vivo, and realized the simultaneous detection of COX-2 and ROS, which provided certain conditions for in vivo research. FQ overcomes the limitations of previous COX-2 probes and realizes in vivo and in vitro detection in living animals, which is of great significance for further clinical research on tumor visualization.

## 3. Fluorescent Probe for Detecting Nitroreductase (NTR) Enzymes in Tumor Cells/Tissues

Hypoxia is characteristic of most advanced tumors. Compared to normal cells, hypoxic cells exhibit a higher level of reductase, such as nitroreductase (NTR) [27,43,44,45,46]. Therefore, NTR can be used as a feasible substance for tumor detection [47,48,49,50]. The degree of hypoxia in tumors can be monitored by detecting the level of NTR. Until now, traditional methods for detecting NTR have been reported, for example, the Clark electrode [51], nuclear magnetic resonance (NMR) [52], and electron paramagnetic resonance (EPR) [53], and so on. However, the above methods have certain limitations, such as complex instruments and low resolution [44]. In contrast, fluorescent probe imaging is low cost, high sensitivity, and simple operation [54,55,56,57]. In recent years, many small-molecule fluorescent probes for NTR have been reported.

**Figure 6:**
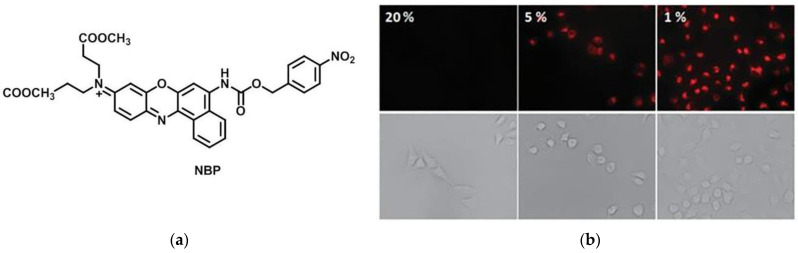
(**a**) Structure of probe NBP; (**b**) In the confocal imaging of A459 cells at different oxygen concentrations. Reproduced with permission from [58]. Copyright 2013 The Royal Society of Chemistry.

In 2013, Qian et al. [58] reported a NIR fluorescence probe (NBP) (Figure 6a), which was synthesized by Nile Blue fluorophore (NBF) and 4-nitrophenyl chloroformate. Under hypoxic conditions, the p-nitrobenzyl of NBP was reduced, so as to release NBF. NBP showed a strong absorption at 525 nm and almost no fluorescence. The fluorophore NBF showed strong absorption and obvious fluorescence emission at 613 nm and 658 nm, respectively. The fluorescence intensity at 658 nm was significantly enhanced when NBP was incubated with NTR. In the confocal imaging of A459 cells, almost no fluorescence was observed under normoxia, while the fluorescence intensity increased obviously when the degree of hypoxia deepened. (Figure 6b) The probe has good selectivity, high sensitivity and low autofluorescence interference, and can be used in tumor diagnosis.

**Figure 7:**
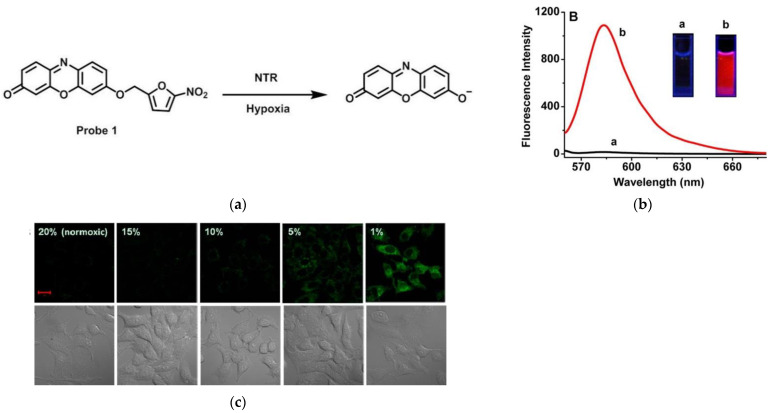
(**a**) Detection mechanism of probe 1 for NTR; (**b**) Fluorescence emission spectra of probe 1 (**b-a**) before and (**b-b**) after reaction with NTR; (**c**) Confocal fluorescence images of Hela cells under different oxygen conditions. Reproduced with permission from [59]. Copyright 2013 American Chemical Society.

Again in 2013, Ma and co-workers [59] described a fluorescent probe 1 (Figure 7a). The probe was introduced into 5-nitrofuran for masking, and resorufin was selected as the signaling unit. When probe 1 reacted with NTR, resorufin was released. Then, the fluorescence of resorufin was recovered (Figure 7b) and it can be seen that a higher level of NTR resulted in stronger fluorescence intensity. Confocal images of Hela cells under different O_2_ conditions (Figure 7c) indicated that the probe can monitor the hypoxic state of the tumor cells by detecting an endo-NTR. This probe is a potential tool to diagnose tumors.

**Figure 8:**
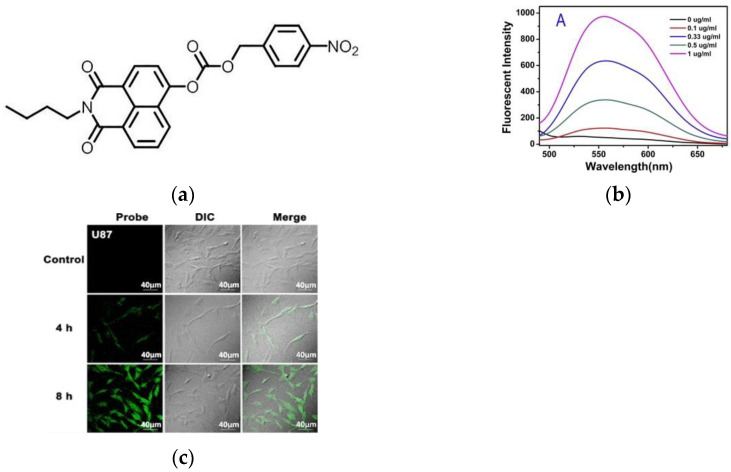
(**a**) Structure of probe 2; (**b**) Fluorescence intensity of the probe reacted in the presence of NTR; (**c**) Laser confocal fluorescence images of U87 cells under the condition of normoxic (20% O_2_) and hypoxic (1% O_2_). Reproduced with permission from [60]. Copyright 2017 Elsevier B.V.

In 2018, Cheng et al. [60] published a novel off–on fluorescent probe 2 (Figure 8a), which was composed by decorating 4-Nitrobenzyl chloroformate moiety with naphthalimide. When the probe reacted with NTR, the fluorescence was released, and as the level of NTR increased from 0.1 mg/mL to 0.33 mg/mL, the fluorescence intensity (Figure 8b) was stronger. As demonstrated in the confocal fluorescence images (Figure 8c) showed that the probe can be applied to detect the hypoxic state of tumor tissue. This probe has high selectivity, low cytotoxicity, and good biocompatibility, and can be utilized for the detection of NTR and imaging of tumor hypoxia.

**Figure 9:**
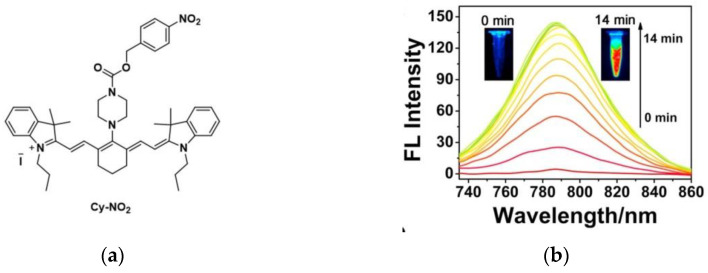

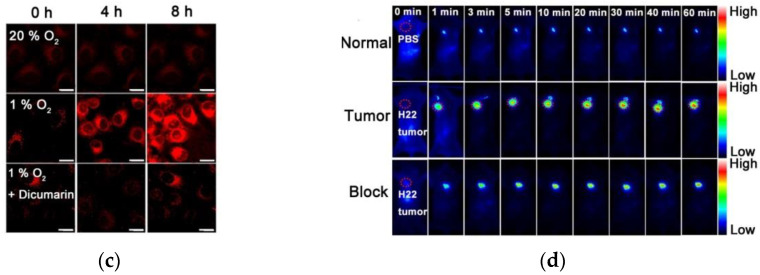
(**a**) Structure of probe Cy-NO_2_; (**b**) Fluorescence spectra of Cy-NO_2_; (**c**)Fluorescence images of A549 cells; (**d**) Fluorescence imaging of Cy-NO_2_ in normal and tumor-bearing mice. Reproduced with permission from [61]. Copyright 2018 Elsevier B.V.

In the same year, Chen et al. [61] developed a NIR fluorescent probe Cy-NO_2_ (Figure 9a). The fluorescence spectrum of Cy-NO_2_ reacting with NTR was shown in Figure 9b, the maximum fluorescence emission occurred at 785 nm. Fluorescence imaging of A549 cells (Figure 9c) showed that Cy-NO_2_ could detect NTR in tumor cells (Dicoumarin is an NTR inhibitor). Furthermore, in vivo imaging of the H22 tumor-bearing mouse model (Figure 9d) showed that Cy-NO_2_ did not fluoresce in normal mice, but strong fluorescence was detected in tumor-bearing mice, whereas the fluorescence intensity increased in mice in the presence of NTR inhibitors smaller. In addition, Cy-NO_2_ was also used in other mouse hypoxia models. Cy-NO_2_ has great research value for tumor hypoxia imaging and imaging of other hypoxia-related diseases.

**Figure 10:**
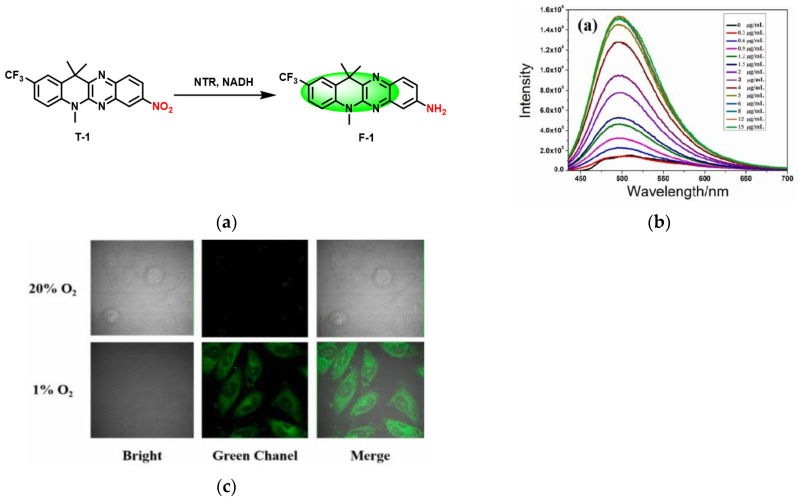
(**a**) Detection mechanism of probe T-1 for NTR; (**b**) Fluorescence spectra changes of probe T-1 upon addition of different concentrations of NTR; (**c**) Confocal images of HeLa cells under different O_2_ conditions incubated with probe T-1. Reproduced with permission from [62]. Copyright 2021 Elsevier B.V.

In 2021, Ji and co-workers [62] designed a “turn on” fluorescent probe (T-1), in which the nitro and trifluoromethyl acted as the electron acceptor, resulting in fluorescence disappearing. Based on the ICT mechanism, NTR reduced the nitro group on T-1 to an amino group, and fluorescence appeared (Figure 10a). When the NTR concentration was 0–6 μg/mL, the fluorescence intensity at 459 nm increased linearly (Figure 10b). T-1 and NTR exhibited stable fluorescence properties in a wide pH range (6–10). As well as confocal imaging (Figure 10c) showed that when the probe T-1 was incubated with HeLa cells, the higher the degree of hypoxia, the stronger the fluorescence. The good membrane penetration, good stability and high selectivity of T-1 allow a good application prospect in the detection of NTR in biological systems.

**Figure 11:**
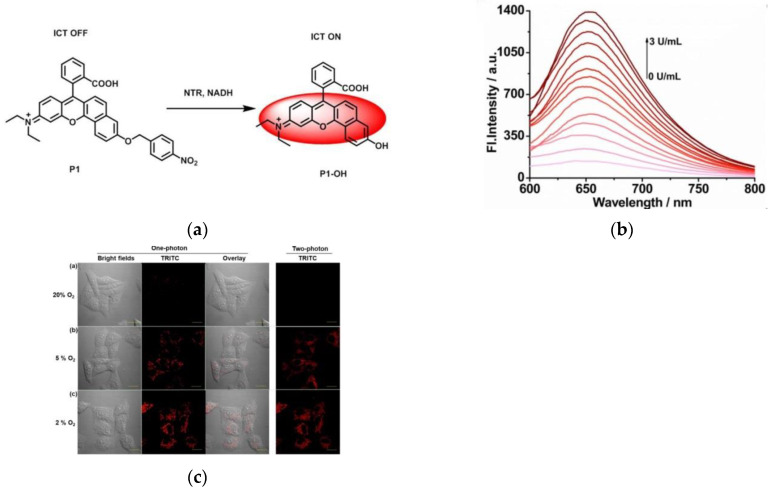
(**a**) Design of probe P1 and its sensing principles; (**b**) Fluorescence spectra of P1 toward series concentrations of NTRs in the being of NADH; (**c**) Fluorescent images for tracing NTRs in HepG2 cells. Reproduced with permission from [63]. Copyright 2021 Elsevier B.V.

In 2022, Lin et al. [63] developed a novel fluorescent probe P1 (Figure 11a). P1 was synthesized based on a two-photon fluorophore P1-OH and NTR recognition site p-nitrobenzene. In the presence of NTRs, the p-nitrobenzene unit of P1 was reduced to -OH, at which point P1-OH exhibited fluorescence emission (Figure 11b) at 647 nm due to the ICT mechanism. HepG2 cells imaging experiments (Figure 11c) showed that after the addition of the probe the fluorescence was weaker in normoxic conditions and had a strong red fluorescence under hypoxia. This probe has a low detection limit and low cytotoxicity. It is of great significance for further identification of cancer cells and tumors.

**Figure 12:**
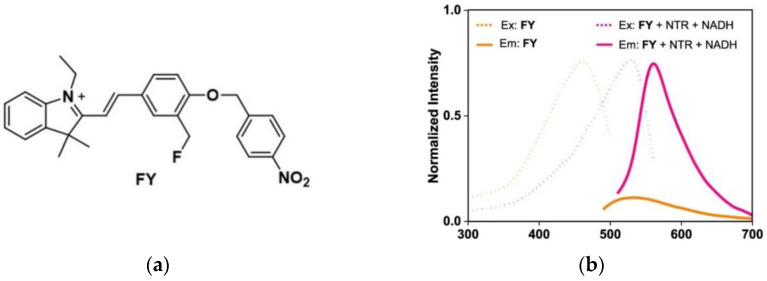

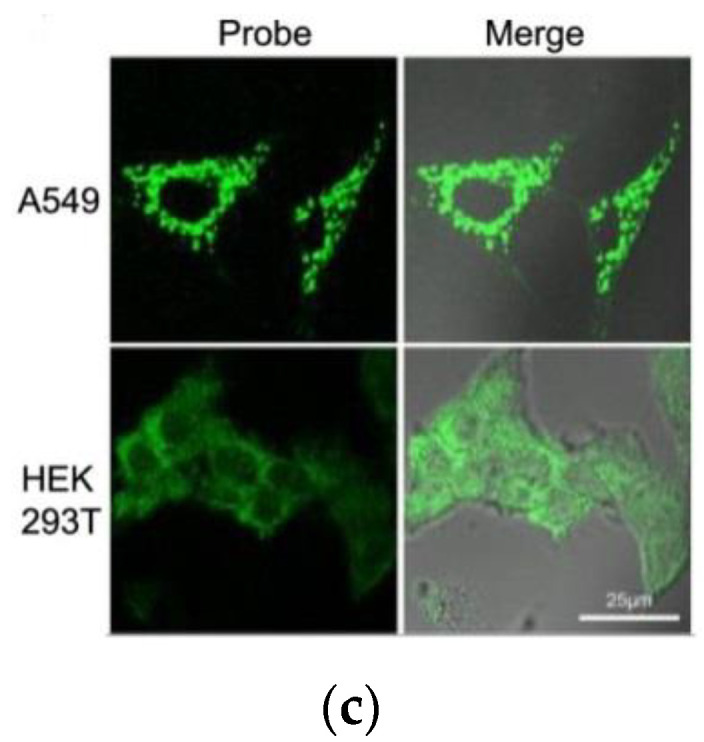
(**a**) Structure of probe FY; (**b**) Spectrogram of NTR and FY reaction in the presence of NADH; (**c**) Fluorescent images of A549 and HEK239T cells. Reproduced with permission from [64]. Copyright 2022 American Chemical Society.

In 2022, Ge et al. [64] reported an NTR-activatable fluorescent probe (FY) in which p-nitrobenzyl was selected as the recognition group of NTR, and hemicyanine was selected as the fluorophore (Figure 12a). Spectral experiments showed that when NTR was added, the fluorescence intensity of the probe changed significantly (Figure 12b). After a series of cell experiments, the results showed that the use of methanol fixation can make the fluorescence signal well preserved, and the fluorescence intensity in A549 cells (cancer cells) is much stronger than that in HEK293T cells (normal cells) (Figure 12c). In addition, FY was successfully used to detect NTR in zebrafish and mice tumor tissues, and the fluorescence signal was found to be more intense under hypoxic conditions. In conclusion, the FY signal can be fixed to better realize the detection of NTR in tumors, which has reference value for further research on biological imaging.

## 4. Fluorescent Probe for Detecting pH

The pH changes in the cytoplasm and some organelles can reflect the state and metabolic processes of cells, especially in some diseases [65,66,67,68]. Studies have shown that one of the features of the environment within malignant tumors is lower pH [65,67,69,70,71,72]. Therefore, the degree of tumor malignancy can be distinguished by detecting intracellular pH. In recent years, due to the low cost, visualization, and high sensitivity [9,73], fluorescent probes for visualization of tumor pH have been reported one after another, which is beneficial to the early diagnosis and treatment of malignant tumors.

**Figure 13:**
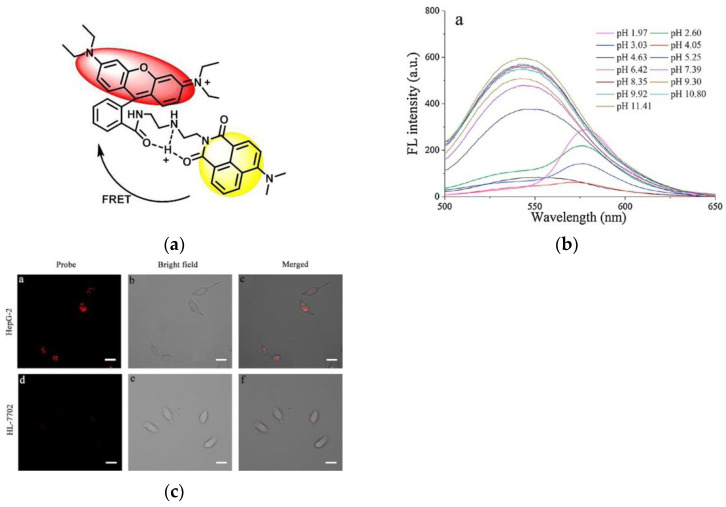
(**a**) The proposed mechanism of RBN for sensing H^+^; (**b**) Fluorescence spectra of RBN at different pH values; (**c**) CLSM images of HepG-2 cells and HL-7702 cells incubated with RBN. Reproduced with permission from [74]. Copyright 2017 Elsevier B.V. on behalf of Chinese Chemical Society and Institute of Materia Medica, Chinese Academy of Medical Sciences.

In 2017, Sun et al. [74] published a naphthalimide–rhodamine-based fluorescent probe (RBN). The probe was based on the FRET mechanism (Figure 13a). In neutral and alkaline environments, RBN emitted fluorescence of naphthalimide; under acidic conditions, rhodamine was ring-open, so that the fluorescence was transferred to the rhodamine. Fluorescent spectroscopy (Figure 13b) revealed that under acidic conditions, a new fluorescence response appeared at 577 nm, which was different from that under neutral alkaline conditions. In confocal imaging (Figure 13c), RBN exhibited a strong red fluorescence in HepG-2 cells, but only weakly in normal cells (HL-7702 cells). The probe is able to provide a vital role in cancer diagnosis and treatment.

**Figure 14:**
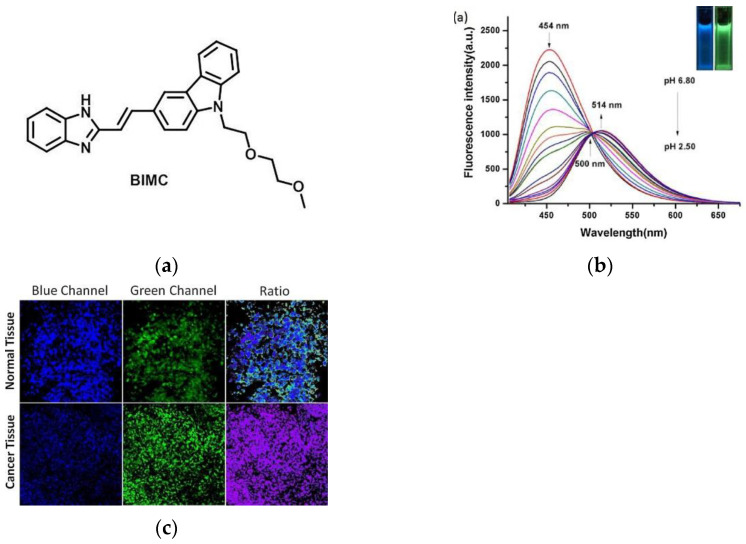
(**a**) Structure of probe BIMC; (**b**) Fluorescence spectra of BIMC with decreasing pH; (**c**) Two-photon ratiometric fluorescence images of pH in mouse liver slices. Reproduced with permission from [75]. Copyright 2018 Elsevier B.V.

In 2018, Wong and co-workers [75] reported a two-photon ratiometric fluorescence probe, BIMC (Figure 14a), which is based on carbazole–benzimidazole. In Figure 14b, when the pH gradually decreased from 6.80–2.50, the fluorescence intensity at 454 nm gradually decreased, the emission band was red-shifted to 514 nm, and an iso-emissive point was formed at 500 nm. The F_454nm_/F_514nm_ had a good linear relationship in the pH range of 5.0–3.82. Hela cells imaging experiments indicated that BIMC can be used as a ratiometric pH probe for cell imaging. Fluorescence imaging of living normal and cancer tissues (Figure 14c) further confirmed the high promise of BIMC in monitoring changes in pH in living tissues. BIMC is of great reference value for the study of lysosome-related pathological and physiological processes.

**Figure 15:**
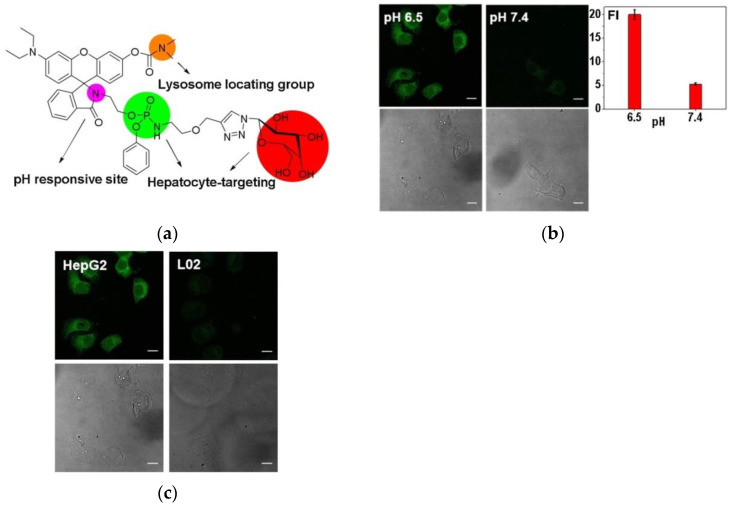
(**a**) Structure of probe HPL-1; (**b**) Fluorescence imaging of HPL-1 in HepG2 cells; (**c**) Fluorescence imaging of HPL-1 in HepG2 cells and L02 cells. Reproduced with permission from [76]. Copyright 2019 Elsevier B.V.

In 2019, Wang et al. [76] developed a new dual hepatocyte-targeting fluorescent probe HPL-1. Figure 15a showed that acid conditions induced internal amide transfer of the rhotamine from closed loop to open loop, resulting in enhanced fluorescence. Cell imaging of HPL-1 under weak acid and alkaline conditions (Figure 15b) revealed that the fluorescence intensity at pH 6.5 was approximately four times that at pH 7.4. Cell imaging experiments of L02 cells and HepG2 cells (Figure 15c) indicated that the probe could precisely distinguish cancerous liver cells from normal liver cells. HPL-1 is important for the precise diagnosis and treatment of hepatocellular carcinoma.

**Figure 16:**
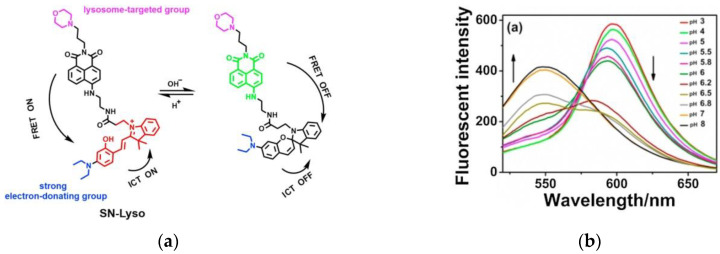

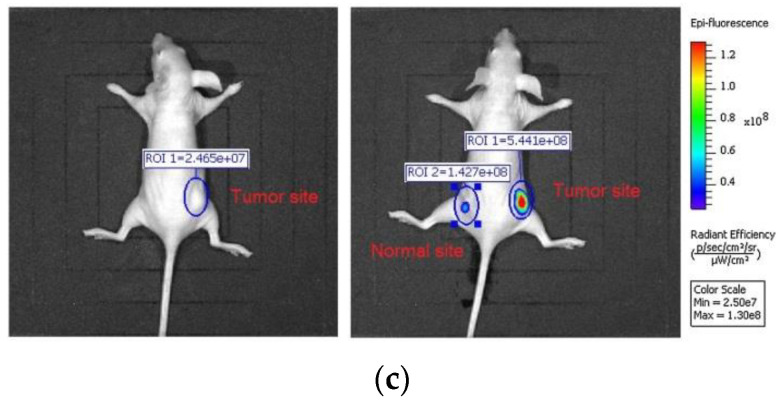
(**a**) Response mechanism of SN-Lyso for pH detection; (**b**) Fluorescence spectra of SN-Lyso at different pH values; **(c**) Fluorescence imaging of the tumor and the normal site in HeLa tumor-bearing nude mice. Reproduced with permission from [77]. Copyright 2021 Elsevier B.V.

In 2021, Yin et al. [77] introduced a new ratiometric fluorescent probe (SN-Lyso) (Figure 16a), which based on the ICT-FRET dual mechanism and morpholine was used as a lysosome-targeted moiety. When the pH was changed from 3 to 8, the fluorescence intensity (Figure 16b) of the probe declined at 597 nm and rose at 550 nm. Cellular imaging of SN-Lyso showed that in HeLa cells and HepG2 cells, the probe could localize to lysosomes and the pH of the lysosomes showed a downward trend during autophagy. Moreover, imaging of the tumor site in mice models (Figure 16c) indicated that the tumor microenvironment in the mice model was more acidic than in the normal microenvironment. SN-Lyso can specifically identify tumor cells, which has a certain reference value for studying the pathological mechanism of lysosomes and visualizing tumor sites.

**Figure 17:**
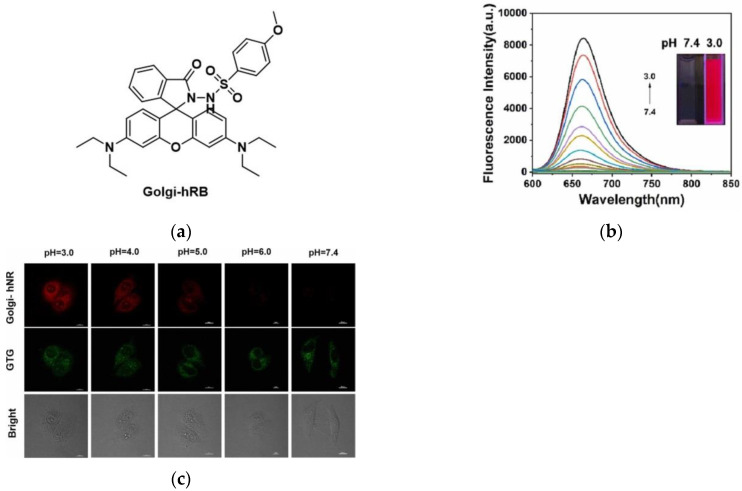
(**a**) Structure of Golgi-hNR; (**b**) Fluorescence spectra of Golgi-hNR; **(c**) Imaging of HepG-2 cells in different acidic environments. Reproduced with permission from [78]. Copyright 2022 Elsevier B.V.

In 2022, Wang et al. [78] synthesized a fluorescent probe (Golgi-hNR) for detecting pH in the Golgi, which chose a rhodamine derivative as the fluorophore (Figure 17a). Spectroscopic experiments showed a trend of fluorescence enhancement at 615 nm from pH = 7.4 to pH = 3.0 (Figure 17b). Golgi-hNR can be selectively localized in the Golgi in HepG-2, HeLa and A549 cells. As shown in Figure 17c, in pH-dependent experiments, the red fluorescence gradually decreased with the increase in pH. In subsequent tests, it was further confirmed that Golgi-hNR can sensitively detect pH changes in the Golgi. In summary, Golgi-hNR can be used to monitor the pH homeostasis of the Golgi apparatus, which is of great significance for the study of diseases such as cancer.

## 5. Fluorescent Probe for Detecting Glutathione (GSH)

Glutathione (GSH) plays an antioxidant role in the redox stress response of living cells, and abnormal GSH content will induce some diseases, such as cancer, Alzheimer’s disease, heart problems, and so on [79,80,81,82,83,84]. Compared with normal cells, GSH is highly expressed in tumor cells for resisting intrinsic oxidative stress [85,86]. GSH as a tumor marker was confirmed in many studies, so the development of GSH probes for tumor recognition is important in the early diagnosis and treatment of tumors [87,88,89,90]. At present, many fluorescent probes have been developed to detect GSH in living cells.

**Figure 18:**
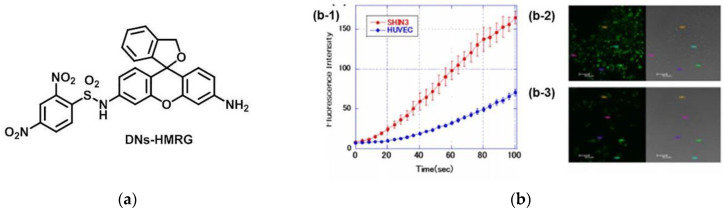
(**a**) Structure of probe DNs-HMRG; (**b**) (**b-1**) Time course of fluorescence intensity of SHIN3 cells and HUVEC cells after adding DNs-HMRG; (**b-2**) Confocal images of SHIN3 cells and (**b-3**) HUVEC cells after adding DNs-HMRG. Reproduced with permission from [82]. Copyright 2014 Elsevier Ltd.

In 2014, Urano et al. [82] published a new fluorescent probe (DNs-HMRG) for GSH (Figure 18a). When the probe reacted with the GSH, the sulfonyl amide bond is rapidly broken and emitted strong fluorescence. Furthermore, the fluorescence response increases with increasing GSH in the physiological level range. In confocal imaging (Figure 18b), DNs-HMRG was co-incubated with SHIN3 cells (GSH-high), and the fluorescence increased, and the fluorescence was aggregated in lysosomes. However, when DNs-HMR was co-incubated with HUVEC cells (GSH-low), and the fluorescence intensity was significantly different. According to intraperitoneal cancer dissemination of SHIN-3 ovarian cancer in a model mouse, DNs-HMRG can be used to detect the dissemination of tiny cancer nodules less than 1 mm in diameter in the abdominal cavity. This probe can be a powerful tool for the study of redox biology.

**Figure 19:**
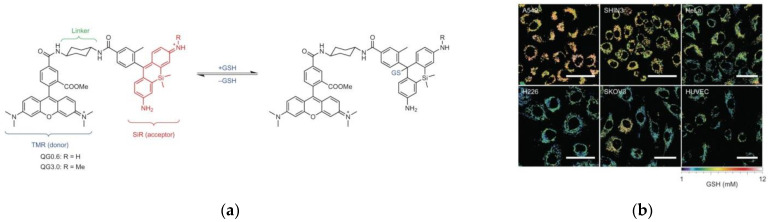
(**a**) Mechanism of QuicGSH activation by GSH; (**b**) Quantification of GSH in several cell lines. Reproduced with permission from [86]. Copyright 2016 Nature.

In 2017, Urano et al. [86] synthesized a FRET-based ratiometric probe, QuicGSH. As shown in Figure 19a, rhodamine and SiR fluorophore were chosen as the fluorophore and a suitable scaffold for developing GSH probes, respectively, and TMR was chosen as the donor. QG0.6 and QG3.0 were synthesized and QG3.0 was used for further study. Further study found that different cell lines exhibited different fluorescence intensities (Figure 19b), which were derived from different GSH concentrations. Experiments showed that QG3.0 can be used to visualize and quantitatively detect intracellular GSH levels, which is of great significance for studying the pathological process of cancer cells.

**Figure 20:**
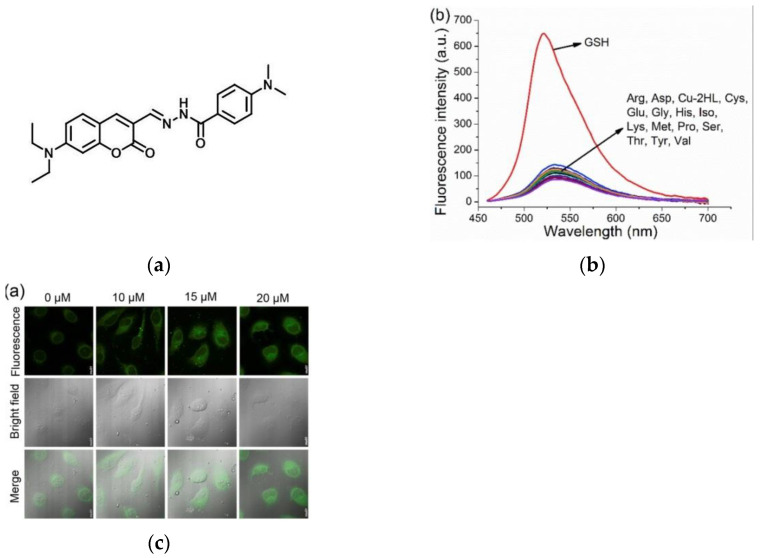
(**a**) Structure of probe HL; (**b**)The fluorescence emission spectra of Cu^2+^-2H; (**c**) Confocal microscope images of MCF-7 cells. Reproduced with permission from [91]. Copyright 2020 Elsevier Ltd.

In 2020, Xu et al. [91] designed a new fluorescent probe, HL (Figure 20a), based on the “on-off-on” fluorescent switch strategy. When HL was combined with Cu^2+^, a new probe Cu^2+^-2HL was synthesized, which had little effect on the fluorescence emission with other amino acids and anions, but the fluorescence emission (Figure 20b) at 521 nm was obviously increased after adding GSH. Confocal microscopy images (Figure 20c) of MCF-7 cells showed that Cu^2+^-2HL could sensitively detect endogenous GSH. MCF-7 cells and HUVEC cells were incubated with Cu^2+^-2HL and imaged under the same conditions. This result indicated that the fluorescence intensity of MCF-7 cells was more than two times stronger than that of HUVEC cells, indicating a more content of GSH in tumor cells. Cu^2+^-2HL and HL have low cytotoxicity, which is of great significance for the detection and recognition of tumors in the life system.

**Figure 21:**
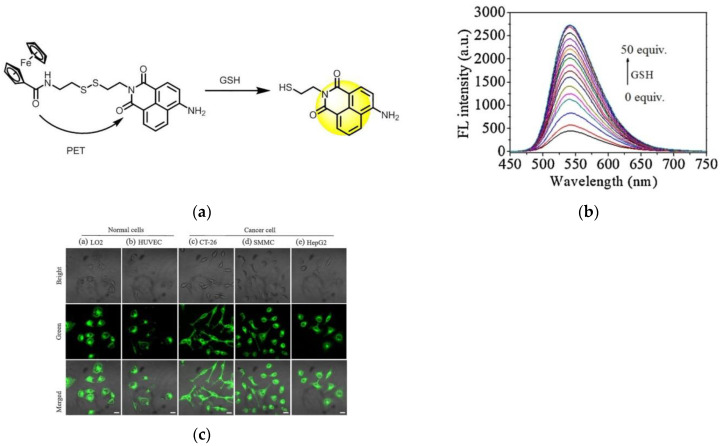
(**a**) Mechanism of Naph-SS-Fc activation by GSH; (**b**) The GSH concentration-dependent fluorescence spectra; (**c**) The CLSM imaging of cells after incubating with Naph-SS-Fc. Reproduced with permission from [92]. Copyright 2020 Elsevier B.V. on behalf of Chinese Chemical Society and Institute of Materia Medica, Chinese Academy of Medical Sciences.

In 2021, Chen and co-workers [92] reported a PET-based fluorescent probe Naph-SS-Fc (Figure 21a). The probe used a disulfide bond as a connecting group, one end was connected to a naphthalimide fluorophore, and the other end was connected to a ferrocene unit. When Naph-SS-Fc reacted with GSH, the disulfide bond was broken, the PET was blocked, and the fluorescence was enhanced (Figure 21b). Confocal imaging showed that Naph-SS-Fc could detect GSH levels in living cells. Two normal cells (HUVEC, LO2) and three cancer cells (SMMC, CT-26 and HepG2) were selected and incubated with the probe, and the results (Figure 21c) showed that the fluorescence intensity of cancer cells was stronger than that of normal cells. Naph-SS-Fc has low cytotoxicity, can be used to detect GSH in living systems, and can distinguish cancer cells from normal cells.

**Figure 22:**
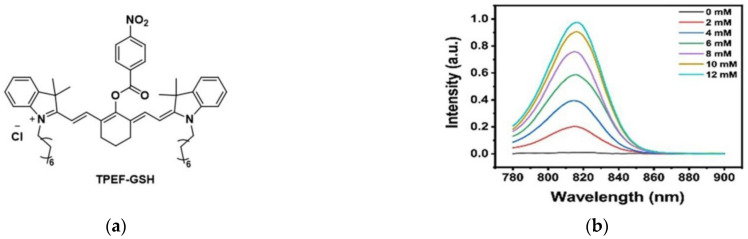

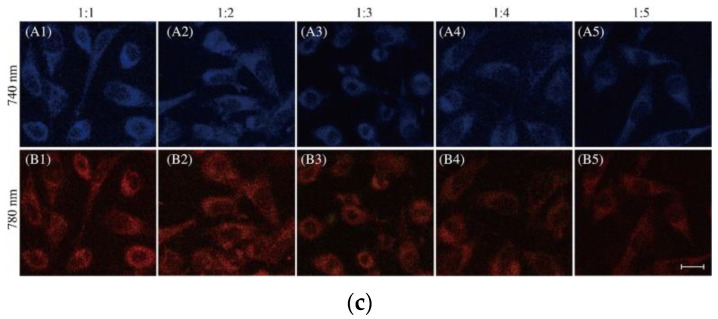
(**a**) Structure of TPEF-GSH; (**b**) The GSH concentration-dependent fluorescence spectra; (**c**) Fluorescence imaging of HeLa cells under different GSH concentrations (GSH:PBS = 1:1–1:5). Reproduced with permission from [93]. Copyright 2022 Wiley-VCH GmbH.

In 2022, Song et al. [93] developed a novel two-photon fluorescent probe TPEF-GSH to detect GSH (Figure 22a). As shown in Figure 22b, the fluorescence intensity gradually increased with the addition of GSH (0–12 mM) at 815 nm. Subsequently, cell experiments were carried out using HeLa cells; it was found that TPEF-GSH could be used to sensitively detect GSH in cells, and cells with high GSH concentration showed more obvious fluorescence intensity (Figure 22c). In addition, zebrafish were used as the in vivo imaging model for a series of studies, the results showed that TPEF-GSH could accurately detect and quantify GSH in tumors. In conclusion, TPEF-GSH is a novel two-photon probe that can image and quantify GSH in vivo and in vitro and has a certain value for the study of two-photon probes in GSH detection in tumors.

## 6. Fluorescent Probe for Detecting Other Biothiols

Small molecule biothiols include GSH, Cys, and Hcy, besides GSH, other small-molecule biothiols also play important roles in cellular operations and signaling [94,95,96,97,98]. The abnormal content of biological thiols in cells may induce some diseases, such as Alzheimer’s disease, malignant tumor, and cardiovascular disease [99,100,101,102,103]. Therefore, the development of probes for rapid and efficient detection of biothiols is of great value for the discovery and therapy of related diseases.

**Figure 23:**
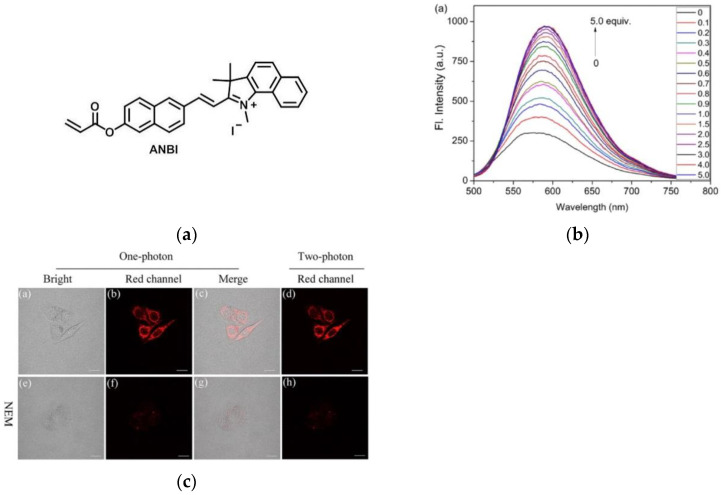
(**a**) The structure of probe ANBI; (**b**) Fluorescence spectra of ANBI with Cys; (**c**) Fluorescence images of HeLa cells. Reproduced with permission from [104]. Copyright 2017 Elsevier Ltd.

In 2017, Lin et al. [104] synthesized a novel two-photon fluorescent probe ANBI (Figure 23a). When Cys (0–10 mM) was gradually added to the probe’s PBS solution, the emission intensity at 590 nm increased linearly (Figure 23b). In Figure 23c, HeLa cells were observed with strong fluorescence after incubation with ANBI. As a control, when HeLa cells were incubated with NEM reagent and then incubated with ANBI, the red fluorescence was weak, indicating that the probe had a strong fluorescence response to intracellular biothiols. Furthermore, tissue imaging experiments showed that the probe can be used to image biothiols in liver and tumor tissues. ANBI has a large Stokes shift, which is conducive to better fluorescence imaging, and has a certain application value for the detection of biological thiols in HeLa cells, liver tissue and tumor tissues.

**Figure 24:**
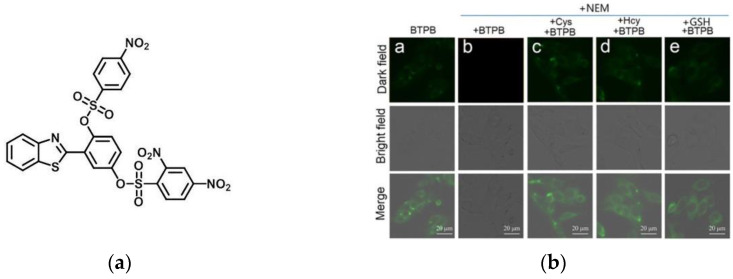
(**a**) The structure of probe BTPB; (**b**) Fluorescence imaging of biothiols in living HepG2 cells. Reproduced with permission from [105]. Copyright 2019 Elsevier B.V.

In 2019, Li et al. [105] reported a ratiometric fluorescence probe (BTPB) (Figure 24a), benzothiazole was selected as the fluorescent precursor. After adding biothiols to the probe solution, the fluorescence at 510 nm appeared to increase gradually. As shown in Figure 24b, the probe exhibited strong green fluorescence after entering HepG2 cells. After adding the probe to the HepG2 cells incubated with NEM, it was found that the fluorescence was weak; after adding the exogenous biothiols, a strong green fluorescence appeared. BTPB has high selectivity and was successfully used to image biothiols in human hepatoma cells and zebrafish, which has certain significance for monitoring biological thiol levels in the cancer cells of organisms.

**Figure 25:**
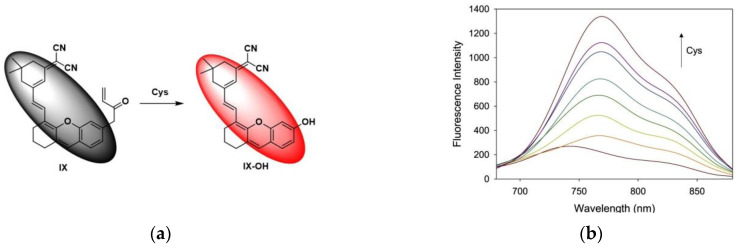

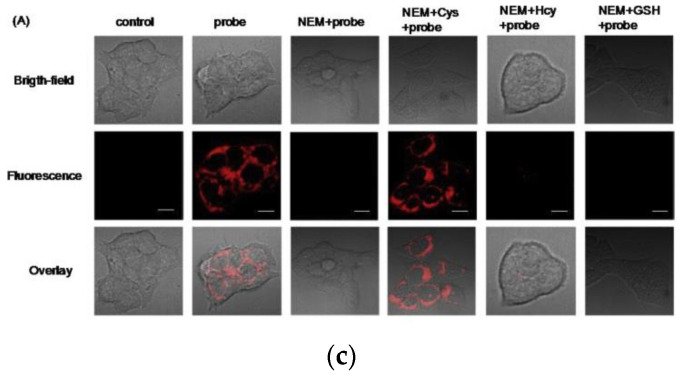
(**a**) Mechanism of the IX activation by Cys; (**b**) Fluorescence spectra of IX; (**c**) Fluorescence imaging of biothiols in HCT116 cells. Reproduced with permission from [106]. Copyright 2021 Elsevier B.V.

In 2021, Li et al. [106] reported a near-infrared fluorescent probe IX (Figure 25a). When IX reacted with Cys, the fluorophore IX-OH was released. As shown in the fluorescence spectrum (Figure 25b), probe IX had a weak emission at 743 nm, and after adding Cys, a new peak appeared at 770 nm. Fluorescence imaging of HCT116 cells (Figure 25c) showed that the probe could specifically detect intracellular Cys. Taking the HCT116-xenograft tumor mice as a biological model, after injection of the probe, the tumor site showed a fluorescent signal, and the signal gradually increased, while no fluorescent signal was seen in the control group. The results indicated that Cys was overexpressed in tumors. IX has low cytotoxicity and high specificity and has research significance for tumor identification and monitoring.

**Figure 26:**
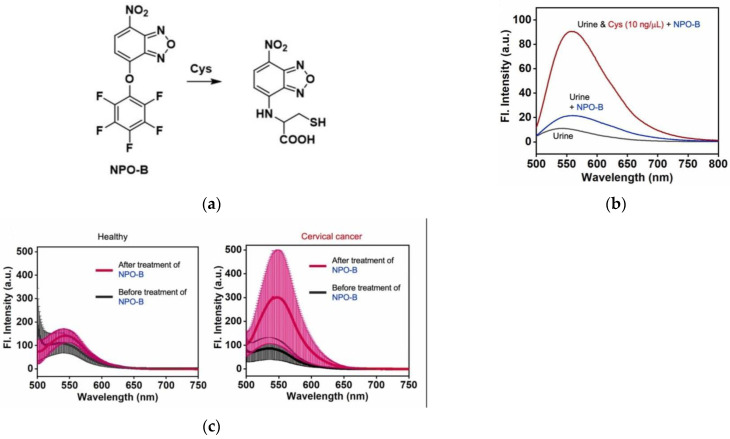
(**a**) Mechanism of the NPO-B activation by Cys; (**b**) Fluorescence spectra of NPO-B; (**c**) Fluorescence spectra of healthy urine and cervical cancer urine. Reproduced with permission from [107]. Copyright 2022 Elsevier B.V.

In 2022, Kim et al. [107] realized the detection of Cys in the urine of cervical cancer patients using the previously reported fluorescent probe NPO-B (Figure 26a). In this work, the healthy control group, urological disorder group and non-urological disorder group were selected as urine samples. As shown in Figure 26b, healthy urine showed no fluorescence, and the fluorescence intensity was weak after NPO-B was added to the urine, while an increase in the fluorescence intensity can be clearly observed after adding Cys. Further, the diseased group was studied, and the analysis results (Figure 26c) showed that compared with the healthy group, the fluorescence intensity of the cervical cancer group increased significantly after adding NPO-B. The urine of cervical cancer patients was further treated with NEM for comparison and it was found that the fluorescence intensity decreased significantly compared with the untreated, indicating that NPO-B can be used to specifically detect Cys in the urine of cervical cancer patients. This is the world’s first method for diagnosing cervical cancer through in vitro diagnostic technology and has a wide range of application values for the diagnosis of cervical cancer.

## 7. Fluorescent Probe for Detecting Hydrogen Sulfide (H_2_S)

Hydrogen sulfide (H_2_S) is a biologically active gas and is considered a gas transmitter, along with nitric oxide (NO) and carbon monoxide (CO) [108,109,110,111]. It has vital functions in some physiological and pathological processes of biological systems, for example, regulating neuronal transmission, modulating insulin release, with a reduced metabolic rate and so on [112,113,114]. Once the intracellular H_2_S cannot be maintained at the level within the physiological range, it will induce diseases such as Alzheimer’s disease, Down syndrome, and other psychiatric disorders [115,116]. In recent years, many studies have found that H_2_S plays a significant role in the growth and proliferation of tumors [117]. H_2_S is a double-edged sword, on the one hand, intracellular H_2_S would induce cell cycle acceleration, activate the migration of tumor cells and invasion, and enhance tumor angiogenesis; on the other hand, high levels of H_2_S are able to control tumor progression and migration and exert antitumor effects [118,119,120].

**Figure 27:**
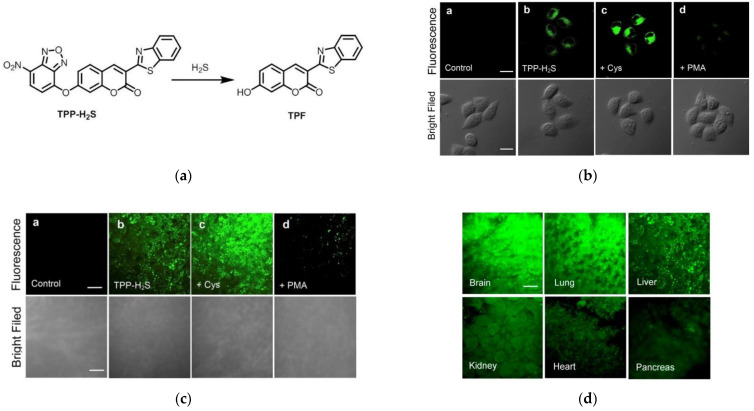
(**a**) Response mechanism of TPP-H_2_S to H_2_S; (**b**) Two-photon confocal microscopy fluorescence images of endo-H_2_S in living HeLa cells; (**c**) Two-photon confocal microscopy fluorescence images of fresh liver slices; (**d**) Two-photon confocal microscopy fluorescence images of different viscera slices. Reproduced with permission from [11]. Copyright 2015 Elsevier B.V.

In 2015, Yang et al. [11] reported a two-photon fluorescent probe (TPP-H_2_S) (Figure 27a). TPP-H_2_S introduced the H_2_S-special recognition group of nitrobenzofurazan in the fluorophore TPF. TPP-H_2_S reacted with H_2_S to release the fluorophore TPF. Significantly, the pure TPP-H_2_S solution had little fluorescence emission, however, in the presence of H_2_S, an approximately 125-fold increase in fluorescence intensity was observed at 490 nm. In confocal imaging (Figure 27b), fluorescence imaging experiments were carried out in HeLa cells. HeLa cells treated with TPP-H_2_S and PMA showed a weak fluorescent response. However, incubation of Cys-treated cells with TPP-H_2_S produced a stronger fluorescence signal. The above results indicated that the probe can detect endogenous H_2_S. Further, exogenous experiments showed that the probe has good membrane permeability. Imaging of H_2_S in rat organ slices (Figure 27c) revealed that TPP-H_2_S allowed deep imaging of H_2_S in tissues. The same phenomenon (Figure 27d) was observed in other organs of the mouse. Hence, TPP-H_2_S provided important implications for the study of the H_2_S-related biological and pathological functions.

**Figure 28:**
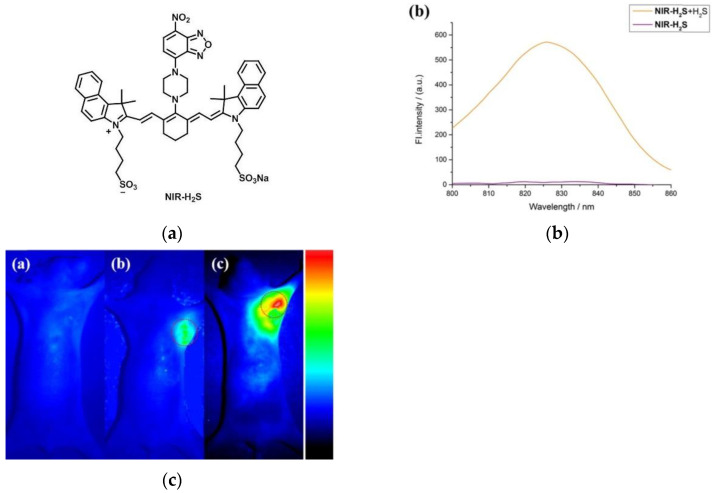
(**a**) Structure of probe NIR-H_2_S; (**b**) Fluorescence spectra of probe NIR-H_2_S; (**c**) Fluorescence images of the tumor-bearing nude mice (**c-a**) The normal, (**c-b**) the HepG2 tumor-bearing and (**c-c**) the MCF-7 tumor-bearing nude mouse was injected with NIR-H_2_S. Reproduced with permission from [121]. Copyright 2018 Elsevier B.V.

In 2018, Wang et al. [121] proposed a Cyanine-based NIR fluorescent probe, NIR-H_2_S (Figure 28a). In the fluorescence spectrum (Figure 28b), NIR-H_2_S showed obvious fluorescence emission at 830 nm after adding H_2_S, and the H_2_S concentration (0–200 μM) had a linear relationship with the fluorescence intensity at 830 nm. When NIR-H_2_S was incubated with MCF-7 cells, only weak fluorescence was exhibited, and the fluorescence was significantly enhanced after adding NaHS. D-Cys and MCF-7 cells were incubated and also fluoresced strongly after adding the probe. The results showed that the probe can detect both endogenous and exogenous H_2_S. In a control experiment (Figure 28c) between the tumor-bearing nude mice (HepG2, MCF-7) and normal nude mice, it was found that the probe NIR-H_2_S has the potential to diagnose H_2_S-related cancers. NIR-H_2_S plays an important role in the diagnosis of some malignant tumors.

**Figure 29:**
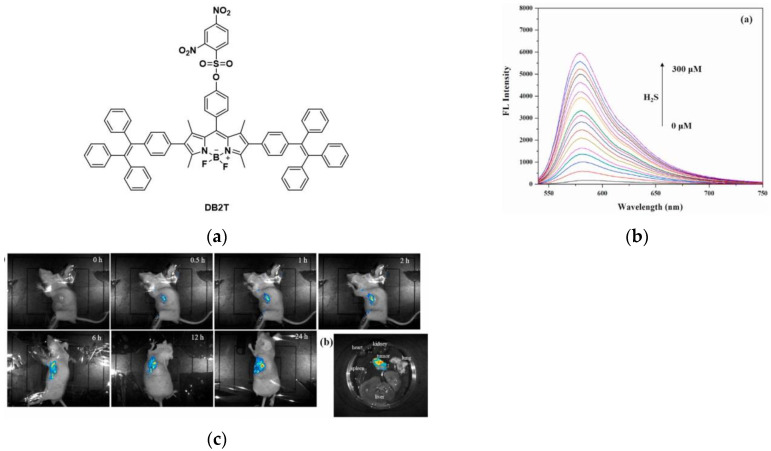
(**a**) Structure of probe DB2T; (**b**) fluorescence response of DB2T; (**c**) In vivo fluorescence imaging of mice bearing HCT116 tumor as well as the major organs and tumor tissue from mice. Reproduced with permission from [122]. Copyright 2021 Elsevier Ltd.

In 2022, Ye et al. [122] reported a BODIPY-based fluorescent probe, DB2T (Figure 29a). When H_2_S was added to the THF/PBS solvent system of the probe, a clear fluorescence “turn-on” response appeared at 579 nm (Figure 29b). Confocal imaging of HCT116, HepG2, PC12 and HUH-7D cells showed that DB2T could fluoresce strongly in H_2_S-enriched cancer cells. Fluorescence imaging in HCT116-tumor-bearing nude mice (Figure 29c) showed that DB2T could effectively accumulate in tumor tissues and exhibited relatively strong fluorescence intensity. DB2T has high selectivity for H_2_S and low cytotoxicity and can be applied to the imaging of H_2_S in tumor cells/tissues, which is of great significance for the diagnosis and treatment of hydrogen sulfide-related malignant tumors.

**Figure 30:**
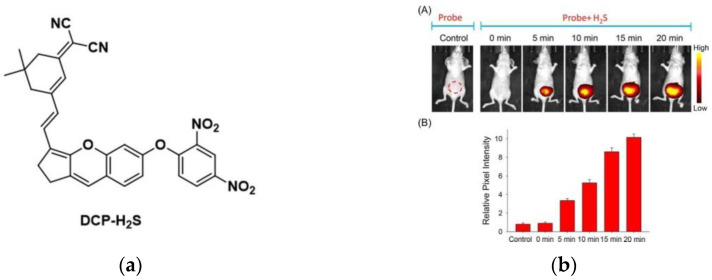
(**a**) Structure of probe DCP-H_2_S; (**b**) Fluorescence imaging of DCP-H_2_S in normal and tumor-bearing mice. Reproduced with permission from [123]. Copyright 2022 American Chemical Society.

In 2022, Li et al. [123] synthesized a NIR fluorescent probe (DCP-H_2_S) in which 2,4-dinitrophenyl was used as the recognition group and isophorone-xanthene was used as the fluorophore (Figure 30a). The PBS buffer of DCP-H_2_S showed very weak fluorescence, and the fluorescence was significantly enhanced after the addition of H_2_S. Further, 239T, Caco-2 and CT-26 cells were studied in cell imaging, the results showed that DCP-H_2_S can detect exogenous and endogenous H_2_S and can distinguish normal cells from cancer cells. In addition, mice imaging was performed. In Figure 30b, DCP-H_2_S showed weakly fluorescent in normal mice while strongly fluorescent in tumor-bearing mice. In conclusion, DCP-H_2_S can not only monitor H_2_S in living cells but also distinguish between normal mice and tumor mice, which will play an important role in cancer diagnosis.

## 8. Fluorescent Probe for Detecting Hydrogen Peroxide (H_2_O_2_)

Reactive oxygen species (ROS) play an important role in maintaining cellular homeostasis and signaling [124,125,126]. Among them, H_2_O_2_ is a kind of ROS and a crucial substance for inducing apoptosis [127,128,129]. Studies have found that H_2_O_2_ is abnormally expressed in some diseases, such as tumors, inflammation, Alzheimer’s disease and other diseases [130,131,132,133,134,135]. Therefore, the monitoring of H_2_O_2_ is of great significance to the diagnosis and treatment of this disease. However, the detection of H_2_O_2_ presents certain challenges due to its short presence [136]. With the development of fluorescence technology, some fluorescent probes for the detection of H_2_O_2_ have been proposed in recent years.

**Figure 31:**
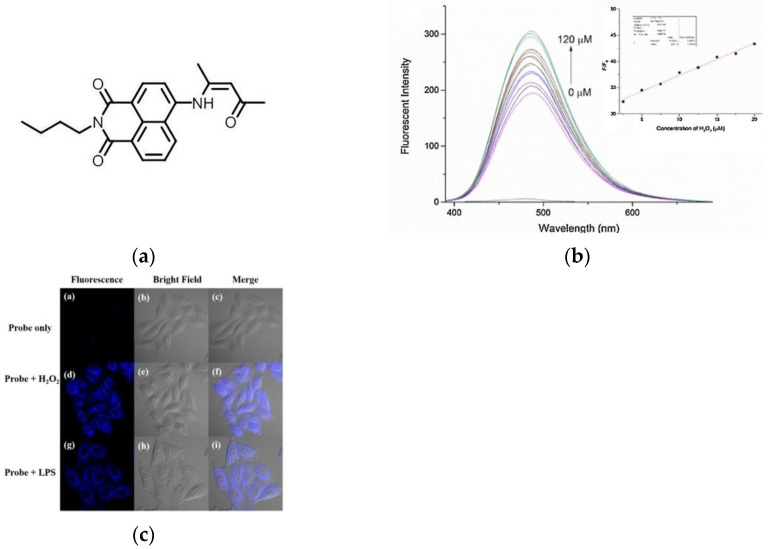
(**a**) Structure of probe GC-2; (**b**) Fluorescence response of GC-2 upon addition of H_2_O_2_; (**c**) One-photon images of HepG2 cells. Reproduced with permission from [137]. Copyright 2017 Elsevier B.V.

In 2018, Wang and co-workers [137] described a fluorescence probe GC-2 (Figure 31a), which is based on ICT. The probe itself had weak fluorescence, and the fluorescence intensity at 485 nm (Figure 31b) was significantly enhanced after binding with H_2_O_2_ and the intensity increased with the increase in H_2_O_2_ level. As shown in (Figure 31c), when HepG2 cells were only incubated with GC-2, there was almost no phenomenon, and obvious blue fluorescence was seen after adding H_2_O_2_. Further, HeLa cells were incubated with lipopolysaccharide (LPS), and a bright fluorescence response was obtained after adding the probe, indicating that GC-2 can detect endogenous and exogenous H_2_O_2_. Based on this, tissue imaging was further explored, and it was found that GC-2 could image endo-H_2_O_2_ in different tumor tissue slices. GC-2 is low toxic, highly sensitive, and can be used for rapid, stable detection of H_2_O_2_.

**Figure 32:**
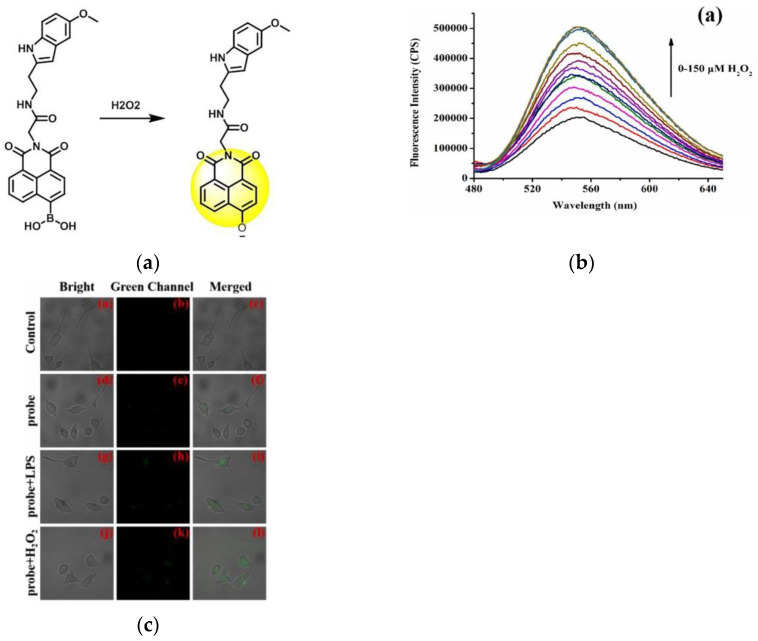
(**a**) Mechanism of the probe activation by H_2_O_2_; (**b**) Fluorescence responses of probe NH-MT toward different concentrations of H_2_O_2_; (**c**) Fluorescence imaging in HepG2 cell. Reproduced with permission from [138]. Copyright 2021 Elsevier B.V.

In 2022, another group (Zhu et al.) [138] published a novel fluorescent probe (NH-MT) (Figure 32a), which used boric acid as a receptor, and naphthalimide was used as a fluorophore to detect exogenous and endogenous H_2_O_2_ in living tumor cells. As shown in (Figure 32b), the fluorescence intensity at 550 nm also increases with the H_2_O_2_ level. As shown in (Figure 32c), there was almost no fluorescence when only NH-MT was incubated, and the fluorescence was enhanced after adding LPS and H_2_O_2_, respectively. Tumor cell lines (MGC803 and HepG2) and normal cell lines (RAW264.7 and HUVEC) were incubated with probes and found that the fluorescence intensity of cancer cell lines was much higher than normal cell lines. NH-MT can target tumor cells and specifically detect H_2_O_2_, providing new ideas for the detection of tumor cells.

**Figure 33:**
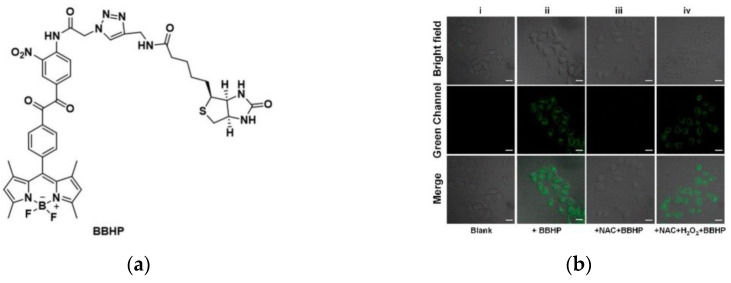

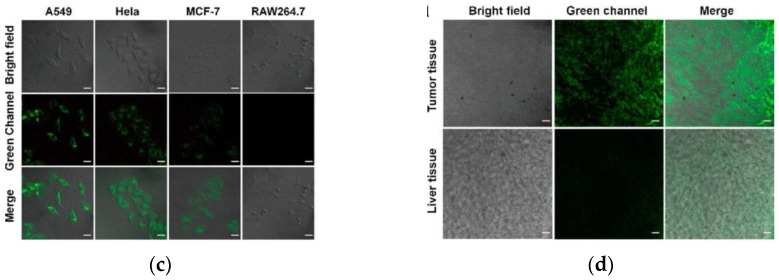
(**a**) Structure of BBHP; (**b**) Fluorescence imaging in HeLa cells; (**c**) Fluorescence imaging in different cancer cells; (**d**) Fluorescence imaging in normal and tumor tissues. Reproduced with permission from [139]. Copyright 2022 American Chemical Society.

In the same year, Duan et al. [139] constructed a fluorescent probe (BBHP) to detect H_2_O_2_ (Figure 33a). BBHP linked with biotin as a cancer cell targeting unit, based on the PET mechanism, released the fluorescence of BODIPY after reacting with H_2_O_2_, also, the fluorescence intensity was enhanced with the increase in H_2_O_2_ concentration. A549, MCF-7, and HeLa cells were used for studies due to biotin receptor overexpression, while biotin receptor-negative RAW264.7 cells were used as controls. Figure 33b showed that BBHP can sensitively detect H_2_O_2_ in HeLa cells (NAC was used to suppress H_2_O_2_ level). Furthermore, Figure 33c demonstrated that BBHP can specifically target cancer cells with overexpressed biotin receptors. More importantly, BBHP was successfully applied to differentiate normal and tumor tissues (Figure 33d), providing a powerful tool for future tumor-specific targeting studies.

## 9. Fluorescent Probe for Detecting Hypochlorous Acid (HOCl)

Hypochlorous acid (HClO) is one of the important ROSs in the living system and is related to many physiological and pathological processes [140,141]. For example, high expression of HClO can lead to risk diseases such as inflammation, cardiovascular disease, tumor, and liver damage [142,143,144,145,146]. Several studies have shown that HclO acid may be abnormally expressed in tumors [147,148,149,150,151,152]. Therefore, monitoring the changes in HclO levels is of great significance in the diagnosis of malignant tumors.

**Figure 34:**
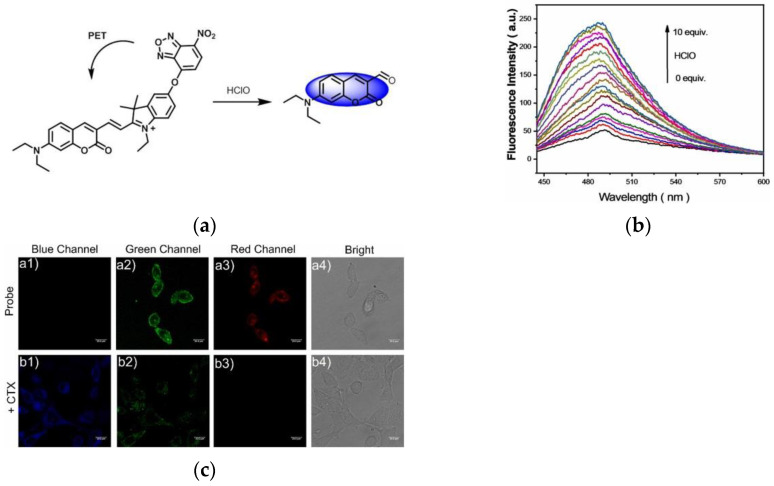
(**a**) Mechanism of the RSS-HClO activation by HClO; (**b**) Fluorescence intensity of RSS-HClO; (**c**) Fluorescence images in CTX-treated KYSE-30 cells. Reproduced with permission from [153]. Copyright 2021 Elsevier B.V.

In 2021, Zhao and co-workers [153] designed a novel fluorescent probe RSS-HclO (Figure 34a), which is based on a coumarin–hemicyanine fluorophore. In the fluorescence spectrum (Figure 34b), with the increase in different concentrations of HClO (0–100 μM), there was a positive linear relationship with the fluorescence emission intensity at 490 nm. Confocal imaging of KYSE-30 cells (Figure 34c) revealed that the probe could visualize HClO in tumors during CTX treatment. Further, in zebrafish imaging, the probe enabled discrimination and detection of HClO. RSS-HClO is low cytotoxicity and good specificity and can be used to detect HClO in tumor cells, which has guiding significance for the treatment of cancer.

**Figure 35:**
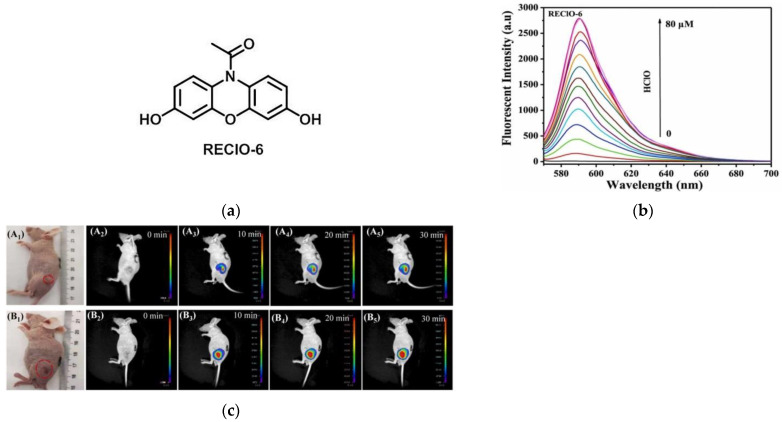
(**a**) The structure of probe REClO-6; (**b**) Fluorescence spectra of the probe REClO-6; (**c**) Fluorescence imaging of mice with different tumor sizes using REClO-6. Reproduced with permission from [154]. Copyright 2021 Elsevier B.V.

In 2022, Wu et al. [154] reported a turn-on fluorescent probe, REClO-6 (Figure 35a). As shown in Figure 35b, when HClO was added to the solution of REClO-6, the fluorescence intensity increased significantly as the concentration of HClO increased from 0 to 50 μM. In confocal imaging, HeLa cells were selected as the bioassay model, and the results showed that the probe can be used to detect the exogenous HClO level in cells. In experiments in mice tumor models (Figure 35c), it was found that REClO-6 was able to emit distinct fluorescent signals at the tumor site. This probe has the ability to rapidly detect HClO and can be applied to solid tumor HClO imaging.

**Figure 36:**
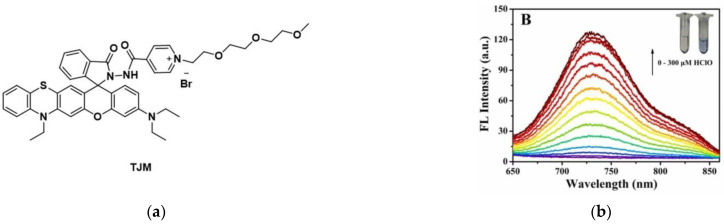

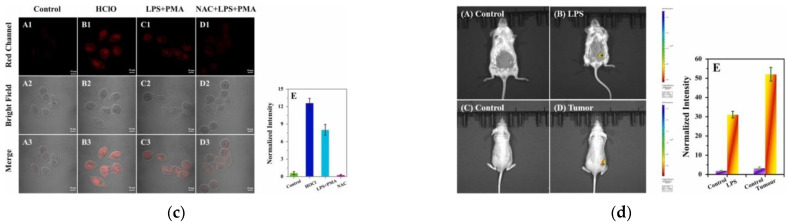
(**a**) Structure of probe TJM; (**b**) Fluorescence spectra of TJM; (**c**) Fluorescence imaging of HeLa cells; (**d**) Fluorescence imaging of mice. Reproduced with permission from [155]. Copyright 2022 Elsevier B.V.

In 2022, Li et al. [155] developed a NIR fluorescent probe, TJM (Figure 36a). TJM emitted strong fluorescence after reacting with HClO, and the fluorescence intensity was proportional to the concentration of HClO (Figure 36b). The results of HeLa cell imaging showed that TJM could detect both endogenous and exogenous HClO in living cancer cells (Figure 36c) (NAC could cause a decrease in HClO). In addition, TJM can be used to detect HClO in zebrafish. Significantly, TJM was found to be useful for the detection of HClO in tumor-bearing mice, which showed that HClO was overexpressed in tumor tissues (Figure 36d). In conclusion, TJM was successfully used to detect HClO in living cells and mice with lesions such as tumors and has a certain reference value in the study of HClO-related diseases such as cancers.

## 10. Fluorescent Probe for Detecting NADH

Enzymes play an extremely important role in the life processes of complex organisms and play an increasingly important role in pathophysiology [156,157,158,159,160]. Studies have shown that abnormal activity of reduced nicotinamide adenine dinucleotide (NADH) and its phosphate NADPH is associated with various diseases, such as diabetes and cancer [161,162,163,164]. It is worth noting that NADH is overexpressed in some malignant tumors and thus can be used as an important substance to distinguish normal cells from malignant cells [165,166].

**Figure 37:**
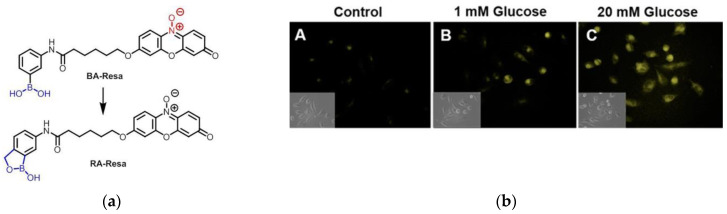
(**a**) The structure of probe BA-Resa; (**b**) Microscopic images of probe RA-Resa in live OSCC cells. Reproduced with permission from [167]. Copyright 2016 American Chemical Society.

In 2016, Chang et al. [167] synthesized a boronic acid-containing fluorescent probe BA-Resa, which was further modified as RA-Resa, for the detection of NADH in living cells (Figure 37a). The fluorescence response of the latter was more pronounced. OSCC cells were selected as model cell lines (Figure 37b). Since intracellular NADH levels were largely affected by glucose concentration, incubation of live cells with glucose and then with probes showed higher fluorescence intensity than those without incubation with glucose, and the results showed that the higher the glucose concentration, the stronger the fluorescence. Equivalent results were shown in human cervical cancer cell lines and CHO cell lines.

**Figure 38:**
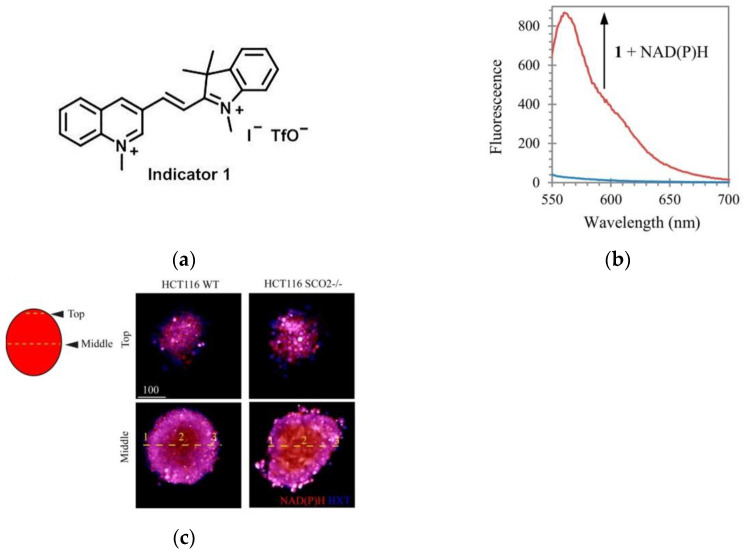
(**a**) The structure of indicator 1; (**b**) Fluorescence spectra of indicator 1; (**c**) Staining of NAD(P)H with indicator 1 in 3D spheroid culture of HCT116 cells. Reproduced with permission from [168]. Copyright 2016 American Chemical Society.

Again in 2016, König et al. [168] designed a novel fluorescent probe 1 (Figure 38a). In Figure 38b, the probe bound to NAD(P)H, resulting in a clear increase in fluorescence intensity at 561 nm. In cell experiments, HEK-293 cell lines were selected, and the results confirmed that the probe has low cytotoxicity and can be applied to the detection of NAD(P)H in mammalian cells. Imaging in the tumor spheroid model (Figure 38c) showed that the probe responded significantly to the fluctuation of NAD(P)H in tumor cells.

**Figure 39:**
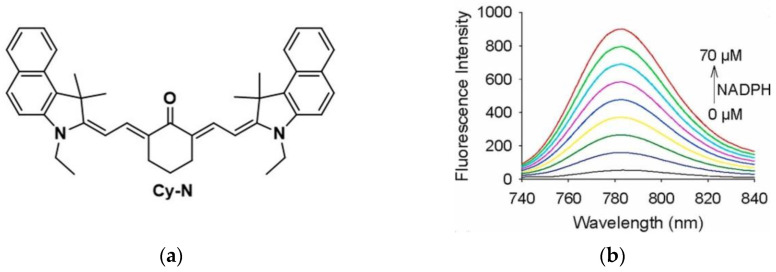

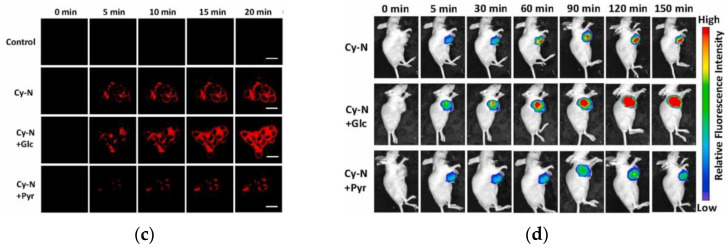
(**a**) Structure of probe Cy-N; (**b**) Fluorescence spectra of Cy-N; (**c**) Fluorescence imaging of NAD(P)H in HCT116 cells; (**d**) Fluorescence imaging of NAD(P)H in tumor-bearing mice. Reproduced with permission from [169]. Copyright 2021 Elsevier Ltd.

In 2021, Li and co-workers [169] reported a multifunction probe (Cy-N) in which cyanine was selected as the fluorophore (Figure 39a). Cy-N had strong fluorescence in the NIR region (783 nm) after reacting with NAD(P)H, and the fluorescence intensity gradually increased (NADPH: 0–70 μM) (Figure 39b). Then, in the cell imaging experiments of various cancer cells (HepG2, HeLa, and 4T1 cells), it was found that Cy-N could detect NAD(P)H in cancer cells. Furthermore, Figure 39c showed that Cy-N can sensitively monitor the changes of NAD(P)H level in HCT116 cells (Glc can promote the high expression of NAD(P)H, and Pyr can reduce NAD(P)H level). More interestingly, Cy-N realized the imaging of NAD(P)H in tumor-bearing mice (Figure 39d), and after dissection, it was found that only tumor tissue had fluorescence and there was no signal in normal organs. The same conclusion was obtained in PA and PTT imaging. In conclusion, Cy-N is a new strategy to detect NAD(P)H in tumor-bearing mice by dual-modal imaging and realizes tumor PTT therapy, which has a certain value for future tumor diagnosis and treatment.

**Figure 40:**
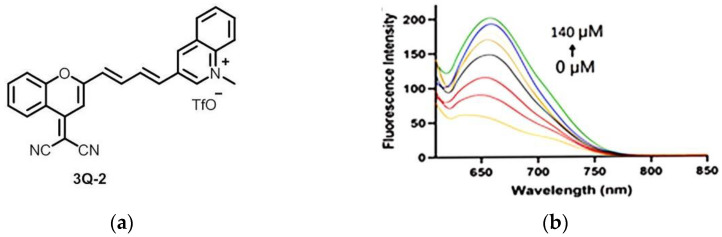

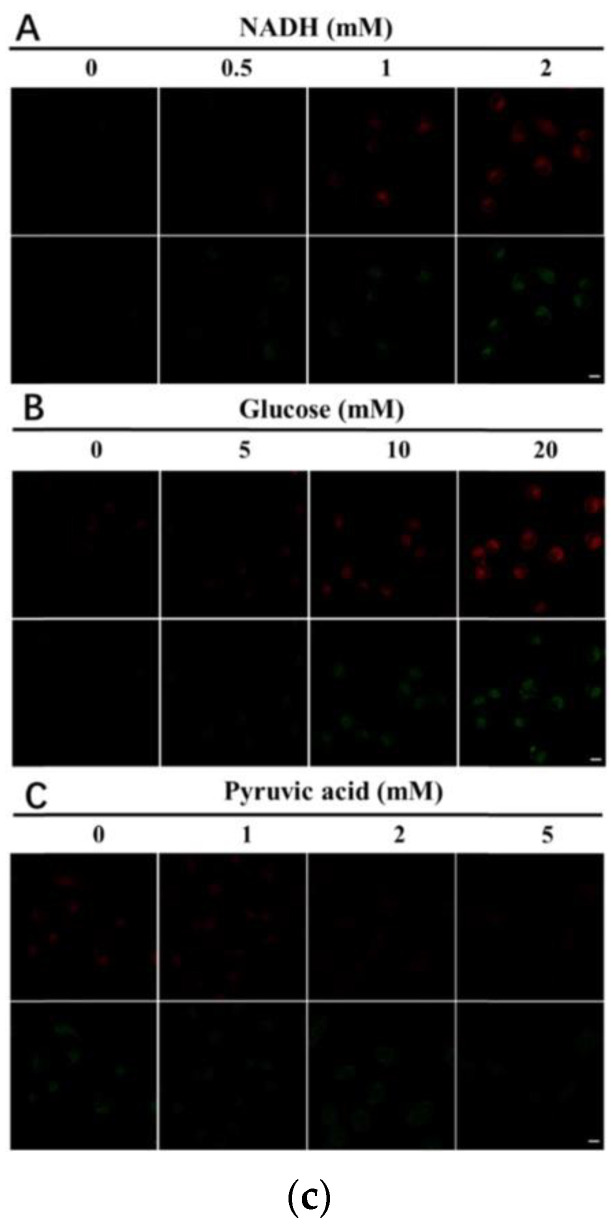
(**a**) The structure of probe 3Q-2; (**b**) Photoluminescence spectra of 3Q-2 incubating with NADH; (**c**) Confocal imaging of 3Q-2-stained PANC-1 cells. Reproduced with permission from [170]. Copyright 2021 Elsevier B.V.

In 2022, Zhang et al. [170] developed a dual-responsive fluorescent probe 3Q-2 (Figure 40a). The probe 3Q-2 exhibited a maximum fluorescence emission at 670 nm, and the emission intensity increased with the increase in NADH concentration (Figure 40b). Fluorescence imaging of 3Q-2 in HT-1080 cells showed that the probe had good membrane permeability and could detect NAD(P)H in the cytoplasm. In the imaging of PANC-1 cells (Figure 40c), exogenous NADH, glucose, and pyruvic acid were used to modulate cytosolic NADH levels, respectively, and the results showed that 3Q-2 could monitor NAD(P)H levels in living cells. The probe enables simultaneous imaging of NAD(P)H and mitochondrial viscosity, and further reveals changes in NAD(P)H during cancer cell ferroptosis, which is expected to be further applied in the detection of NAD(P)H in cancer cells.

## 11. Conclusions

In this review, we summarized the research progress of small-molecule fluorescent probes for detecting some substances abnormally expressed in tumors in recent years. Herein, we start with several different tumor-related substances and briefly introduce the molecular structures, spectral properties, and bioimaging of fluorescent probes. Finally, we hope that this review can encourage researchers to design more excellent fluorescent probes, and provide a certain reference value for the detection of abnormally expressed substances in tumors and then distinguish tumors from normal tissues in the future; we further hope that this review will have a certain impetus for the clinical diagnosis and treatment of tumors in the future.

## Data Availability

Not applicable.
